# Emergent Roles of RVLM^C1^ Neurons in Cardiovascular Homeostasis and Systemic Physiological Regulation

**DOI:** 10.1007/s11906-026-01400-8

**Published:** 2026-11-05

**Authors:** George M.P.R Souza, Oluwaseun S. Faborode, Ana C.M Omoto

**Affiliations:** Department of Physiology and Biophysics, University of Mississippi Medical Center, 2500 N State St, Jackson, 39216 MS USA

**Keywords:** RVLM, Sympathetic activity, Baroreflex, Autonomic nervous system, Presympathetic neurons, State-dependent

## Abstract

**Purpose of the Review:**

Adrenergic C1 neurons in the rostral ventrolateral medulla (RVLM^C1^) have long been recognized for their role in regulating sympathetic activity and arterial blood pressure (BP). However, novel approaches such as genetic targeting, optogenetics, fiber photometry, among others, allowed us to study the biology of these neurons in deeper detail and in non-anesthetized preparations, unraveling previously underappreciated or unrecognized functions. Here we will discuss recent studies that explored the function of RVLM^C1^ neurons for the sympathetic regulation of BP and also the emergent roles of these cells for other processes involved in systemic physiological regulation.

**Recent Findings:**

Using selective methods of manipulation and recording of neuronal activity in conscious animals, it was demonstrated that RVLM^C1^ neurons play a critical role in BP stability across multiple behaviors, including sleep-wake state and postural changes. Besides cardiovascular control, recent evidence shows that these neurons play a critical role in different homeostatic functions such as thermoregulation, immune response, glycemia and in complex behaviors such as anxiety.

**Summary:**

RVLM^C1^ neurons are a key hub that integrates interoceptive signals from peripheral systems to regulate BP and other basic physiological functions. Along with their role in cardiovascular regulation, these emergent roles of RVLM^C1^ neurons may also need to be considered when explaining the complex and multifactorial pathophysiology of cardiometabolic diseases associated with hypertension.

## Introduction

Adequate levels of blood pressure (BP) are necessary for maintaining optimal tissue perfusion. The blood flow requirements vary with metabolic demand, movement and postural changes, requiring a highly dynamic system that adjusts BP accordingly [1–3]. The short-term regulation of BP is designed to prevent sudden drops (hypotension) or exaggerated increase in BP (hypertension) that occurs in the order of seconds during a large variety of mammalian behavior [1–3]. This regulation of BP is not only fast but also highly precise. This is possible due to components of the autonomic nervous system that sense changes in systemic arterial BP beat-to-beat and engages vagal and sympathetic activity to modulate cardiovascular function, with the ultimate goal of buffering large and disproportionate oscillations in BP [2, 3]. Among the autonomic neurons that regulate sympathetic activity, the adrenergic neurons in the rostral ventrolateral medulla, named C1 (RVLM^C1^), have been hypothesized as key elements in the sympathetic regulation of BP [4–6].

RVLM^C1^ neurons are barosensitive, meaning that they are inhibited by increases in systemic BP and stimulated by hypotension [4, 7]. In the past, this fact could be only demonstrated in anesthetized preparations, which precluded the investigation of the activity of these barosensitive neurons across dynamic and complex behaviors that influence BP control [1, 2]. Recent technological advances, such as genetic targeted fiber photometry and optogenetics, allowed not only to record the activity of RVLM^C1^ neurons but also to control their activity during distinct behaviors in conscious preparations. These advances have provided a new perspective on the role of RVLM^C1^ neurons in BP regulation and in the broader coordination of complex autonomic and homeostatic functions, such as metabolic regulation, arousal states and immune function.

Our understanding of RVLM^C1^ neuron function has been built over several decades through a diverse array of experimental preparations and methodological approaches, each contributing important insights into their neurobiology [4]. This review is not intended to provide a historical account of the field. Rather, we focus on recent advances, with particular emphasis on studies conducted in conscious animals and those using increasingly selective approaches to identify and manipulate RVLM^C1^ neurons. We aim to provide an updated perspective on the role of these neurons in the regulation of homeostatic and allostatic states and to discuss how these recent discoveries may advance our understanding of the neural mechanisms contributing to the pathophysiology of hypertension.

## Definition, Targeting and Manipulation of RVLM^C1^ Neurons in Rodents

Classic histological definition of C1 neurons consists of cells in the RVLM that express phenylethanolamine N-methyltransferase (PNMT) and Tyrosine Hydroxylase (TH), enzymes involved in catecholamine synthesis [4, 8, 9]. There are at least two subtypes of RVLM^C1^ neurons, which could be distinguished according to their projections to the spinal cord or hypothalamus [4, 7]. The presympathetic RVLM^C1^ neurons are located more rostrally, are barosensitive and project to the intermediolateral column of the spinal cord [7]. The most caudal RVLM^C1^ group projects mostly to the hypothalamus and are also characterized by the expression the neuropeptide Y [4, 9]. Both subtypes seem to be involved in the control of BP either directly by stimulating preganglionic neurons or indirectly via stimulation of supra medullary autonomic regions [4]. However, the specific function of these distinct subtypes, or if there are still more subclassifications of RVLM^C1^ neurons, needs to be determined.

With the advent of transgenic rodents, it is possible to use the genes for the catecholamine-synthetizing enzymes present in RVLM^C1^ to design viral vectors encoding specific promoters or transgenic rodents carrying Cre-recombinase in TH+, PNMT + or dopamine β-hydroxylase (Dβh+) neurons. Now, Cre-dependent expression of a wide range of genes, including opsins and genetically encoded calcium indicators, enables more selective targeting and functional characterization of these neurons in conscious, freely behaving animals. Advances in intersectional genetic approaches could provide increasingly selective targeting of RVLM^C1^ neurons [10]. Moreover, by monitoring the activity of RVLM^C1^ neurons using genetically targeted fiber photometry in conscious animals is possible to reveal more subtle stimuli or associated behaviors that influence the activity of these neurons. This represents a great advance compared to the strong and often supraphysiological stimulus used to study the activation of these cells via c-Fos expression. Using optogenetic and chemogenetic approaches, these neurons can be selectively stimulated or inhibited in an acute and reversible manner, thereby reducing potential confounding factors in data interpretation. Finally, data of single cell RNA sequencing of RVLM neurons is publicly available and might offer an opportunity to elaborate hypothesis of different subsets based on their molecular profile [11]. By integrating these molecular and functional approaches, we might expect that the definition of RLVM neurons, including RVLM^C1^, is not static and will become more diversified by linking specific neuronal types to a given physiological function.

## RVLM^C1^ Neurons are Necessary for the Short-Term Control of Arterial Blood Pressure

Dynamic behavior as observed in conscious subjects requires constant and precise regulation of blood flow and arterial BP to keep perfusion pressure adequate [2]. The long-term regulation of BP involves several mechanisms, including renal and endocrine adjustments [12, 13] whereas its short-term regulation relies on the sympathetic and vagal components [3]. The RVLM^C1^ neurons have long been recognized by their role in BP control, however, much of the information regarding these neurons either relies on anesthetized preparations and/or in non-selective methods to target these neurons. The implementation of genetically targeted fiber photometry enabled the study of barosensitive RVLM^C1^ neurons activity with high-temporal resolution in conscious animals with simultaneous measurement of BP [14, 15]. This advance provided an opportunity to correlate the activity of RVLM^C1^ neurons and arterial BP during distinct natural behaviors. Using such recordings, it was possible to confirm that the activity of RVLM^C1^ neurons is dynamic and varies according to behavior and sleep-wake cycle, as expected for the sympathetic activity [15, 16]. Specifically, RVLM^C1^ neurons are activated during cortical arousal (spontaneously or induced), rapid-eye movement (REM) sleep and rearing [15]. Their activity progressively reduces, similar to BP and heart rate, as the animal enters in non-rapid eye movement (NREM) sleep. The dynamic activity of RVLM^C1^ neurons is partially shaped by the arterial baroreflex, as sino-aortic denervation attenuates their activation during certain behavioral states, such as REM sleep. In contrast, arousal-related increases in RVLM^C1^ activity appear to be driven primarily by central command, as baroreceptor denervation does not clearly alter this pattern [15].

The functional significance of these dynamic changes in RVLM^C1^ activity for BP control is demonstrated by the effect of the selective​-targeted ablation of these neurons using cre-dependent Caspase3 expression [15]. Importantly, chronic lesions of RVLM^C1^ neurons do not have an impact in the long-term regulation of BP, as the mean arterial pressure and its circadian regulation remain unaltered. However, BP variability is increased after RVLM^C1^ ablation, an effect that is particularly evident during wakefulness and stress-induced arousal from sleep [15]. Moreover, these animals present hypotension during REM sleep and during postural changes such as rearing or jumping over a wall. Altogether, this recent evidence indicates that RVLM^C1^ neurons are activated and necessary for BP stability in a state dependent-manner in freely behaving conditions [15].

## RVLM^C1^ Neurons are Activate by Hypotension and Hemorrhage

Despite being activated during several natural behaviors, the activity of RVLM^C1^ neurons seems to be relatively low during NREM sleep or when the animal is quiescent as measured by fiber photometry [14, 15]. This is consistent with the fact that optogenetic inhibition of RVLM^C1^ neurons produces a relatively small decrease in sympathetic activity and BP in resting conditions [14, 17]. However, their activity is promptly recruited in response to hypotension induced by peripheral vasodilation or hypovolemia induced by controlled hemorrhage in conscious animals [14, 15]. In fact, these neurons are the primary source of sympathetic drive that maintains mean arterial pressure during the compensated phase of hemorrhage [14]. This is evident by the acute optogenetic silencing of these neurons during blood loss, which produces a pronounced sympathetic inhibition and a large drop in BP. As blood loss progresses, hemorrhage enters the so-called decompensated phase, characterized by a reduction in RVLM^C1^ neuron and sympathetic activity, leading to hypotension [14]. This phenomenon seems to be a reflex orchestrated by the autonomic nervous system rather than a consequence of hypovolemia itself, as re-activating RVLM^C1^ neurons using optogenetics can restore BP during decompensated hemorrhage [14].

The activation of RVLM^C1^ neurons by hypotension is mediated by the arterial baroreceptors, as sino-aortic denervation, almost completely eliminates their response to this stimulus [15]. However, a small portion of the response still remains which might indicate that they could also sense a transient cerebral hypoperfusion that might occur during hypotension. The intrinsic sensitivity of RVLM^C1^ neurons to hypoperfusion/hypoxia might have implications for hypertension as it has been hypothesized that persistent cerebral hypoperfusion and brainstem hypoxia contribute to the elevated sympathetic drive in the spontaneously hypertensive rat (SHR) [18]. However, the link between hypoperfusion/hypoxia, RVLM^C1^ neurons and hypertension still need to be determined, especially in conscious conditions.

## RVLM^C1^ Neurons Regulate Arousal State

Optogenetic stimulation of RVLM^C1^ neurons potently drive arousal from NREM sleep along with increases in BP in conscious rats [19–21]. However, these neurons seem to be redundant for the arousal induced by hypercapnia, hypoxia, acoustic stimulation or air-jet [15, 22]. Nevertheless, the ability of RVLM^C1^ neurons to promote arousal may contribute to physiological behaviors associated with high-arousal states, such as anxiety. Recent studies using novel intersectional strategies to selectively target RVLM^C1^ neurons (PNMT+, Dβh+) in conscious mice reveal that the stimulation of these neurons lead to anxiety-like state via their projections to ventral lateral periaqueductal gray neurons (vlPAG) [10]. Moreover, using fiber photometry, the authors demonstrated that these neurons are also activated in anxiolytic-like states in mice. Finally, the importance of RVLM^C1^ neurons to the generation of anxiety was demonstrated by chemogenetic inhibition of these cells, which reduces the anxiety-like behavior [10].

The autonomic responses to anxiety are complex and could involve increase in sympathetic activity and BP which, over time, might increase the risk for developing hypertension [23]. In fact, chronic and trait anxiety are associated with elevated sympathetic activity in humans [24–26]. In rodents, exposure to a predator scent led to anxiety-like behaviors and a greater sensitization to hypertension [27]. This indicates that the neural pathways involved in anxiogenic behavior might influence the state of neurons controlling the cardiovascular system. Future studies should determine whether RVLM^C1^ neurons are the missing link that connects the cardiovascular responses to anxiety.

## Regulation of Metabolism and Glycemia by RVLM^C1^ Neurons

The sympathetic nervous system plays an important role in metabolic regulation; however, the specific neuronal subtypes and neural circuits involved remain incompletely defined. Recent evidence indicates that chemogenetic stimulation of RVLM^C1^ neurons in mice leads to reduction in body temperature, CO_2_ production and O_2_ consumption, indicating a reduction in overall metabolic rate [28, 29]. These neurons seem to be necessary, at least partially, for the full expression of the torpor response induced by fasting in mice, a phenomenon that seems to be mediated by RVLM^C1^ projections to hypothalamic nuclei [29]. These observations might seem, at first glance, to be contradictory to the potent high-arousal state promoted by RVLM^C1^ neurons described in the previous Sect [10]. The study by Fernández-Peña et al. (2026) found that selective activation of RVLM^C1^ neurons (PNMT + and Dβh+) did not induce torpor or freezing, but instead promoted a high-arousal state, consistent with observations from studies in which these neurons were stimulated in rats [19–21]. These divergent effects of stimulating Dβh+ neurons in the RVLM, ranging from high arousal to torpor, could be explained by TH+ neuron heterogeneity or by the fact that using Dβh to target RVLM^C1^ neurons also stimulates a subset of A1 neurons. The arousal effect of stimulating TH+ neurons in the RVLM of Th-Cre rats could be explained by fiber placement, whereby more rostral placement may stimulate a subset of TH+ neurons that are predominantly spinally projecting PNMT+ neurons while sparing A1 cells.

It has been long hypothesized that RVLM^C1^ neurons play a role in the regulation of glycemia, especially a subgroup of these cells located more caudal to the spinally-projecting ones [30–32]. Despite being largely dispensable for glucose homeostasis under resting conditions, ablation of RVLM^C1^ neurons attenuates the sympathetic response to glucoprivation, indicating that these neurons contribute to this adaptive response [31]. In fact, chemogenetic stimulation of RVLM^C1^ neurons in Dβh-Cre mice produces glucose mobilization from the liver, leading to hyperglycemia after two hours of the stimulation [28]. This response occurs via the activation of the adrenergic response in the adrenal gland, an effect that could be important during stressful situations that engage a fight or flight response [28]. In summary, RVLM^C1^ neurons have the potential to regulate metabolic state, promoting hyperglycemia and hypothermia. These responses seem to be useful for the allostatic regulation of the systemic response to stress such as infection or fasting [28, 29, 33]. The divergent effects of RVLM^C1^ neuron stimulation, which can induce either arousal or torpor, may depend on the specific C1 neuronal population recruited and/or the internal state, the physiological context and the nature of the stimuli. Finally, the potential role of RVLM^C1^ neurons in cardiometabolic diseases is still unknown and requires further investigation.

## RVLM^C1^ Neurons Modulate the Immune System

Recent studies reveal a pivotal role for the sympathetic nervous system in the modulation of immune system and anti-inflammatory reflex mainly involving RVLM^C1^ neurons. Anticipated acute optogenetic or chemogenetic stimulation of these cells in DβH-Cre mice leads to protection against kidney [34] and lung injury [35]. The protective effects mediated by C1 neurons in acute kidney injury persist following subdiaphragmatic vagotomy or blockade of corticosterone receptors, indicating that these responses are predominantly mediated by sympathetic modulation, especially the adrenergic response by the adrenal glands and the sympathetic modulation of splenic immune cells [34, 35]. Despite the fact that acute stimulation of RVLM^C1^ neurons could protect for future injuries, chronic stimulation of these neurons seems to have a deleterious effect on the anti-inflammatory response. When RVLM^C1^ neurons are stimulated for 7 days using chemogenetics, the protection against lung injury is significantly dampened along with the effect of their stimulation of the adrenal glands [35]. Accordingly, the authors concluded that the impairment of the anti-inflammatory reflex produced by chronic stimulation of RVLM^C1^ neurons was due to deterioration of the adrenergic response in the adrenal glands [35].

The deleterious effects of chronic RVLM^C1^ neurons stimulation were also observed in the progression of tumor growth in mice [36]. The authors observed an increase in tumor volume during 30 days of chemogenetic stimulation of RVLM^C1^ neurons. In contrast, they found that genetically targeted ablation or chronic chemogenetic inhibition of these neurons impair tumor growth [36]. These effects are mediated by CD8 + lymphocytes, indicating an important role of RVLM^C1^ neurons in the modulation of the immune adaptative response to tumors [36]. In summary, RVLM^C1^ neurons can modulate immune function in response to threats, contributing to an anti-inflammatory reflex. However, chronic or sustained activation of these neurons may impair or exhaust this protective response.

## Breathing Regulation by RVLM^C1^ Neurons

RVLM^C1^ neurons are located in proximity to neurons controlling respiratory function in the brainstem, including central chemoreceptor neurons in the retrotrapezoid nucleus (RTN) and rhythm-generating neurons in the pre-Bötzinger complex [4]. RVLM^C1^ is activated by hypoxia and peripheral chemoreceptor stimulation and has been coined before as oxygen sensors in the brain [37–40]. Optogenetic stimulation of RVLM^C1^ increases respiratory frequency and tidal volume and produces sighs in rodents breathing room air [19–21]. Interestingly, these responses could be also reproduced by stimulating RVLM astrocytes, which indirectly activate RVLM^C1^ neurons [41]. However, these neurons do not seem to be particularly involved in the generation of active expiration (increase in abdominal contractions during expiration), likely due to the lack of significant projections to expiratory premotor neurons of the caudal respiratory group that regulate this aspect of breathing [21]. Despite their potential to increase pulmonary ventilation, chronic selective lesions of RVLM^C1^ neurons do not impact resting minute-ventilation, arterial blood gases, sighs or respiratory responses to hypercapnia or hypoxia [22]. However, RVLM^C1^ neurons are necessary for sympathetic regulation of BP in response to chemoreceptor activation, indicated by the fact that acute optogenetic inhibition of these neurons leads to pronounced hypotension during hypoxia in conscious animals [17]. Thus, RVLM^C1^ neurons do not appear to be necessary for the respiratory response itself but rather seem to contribute to the cardiovascular adjustments that accompany chemoreceptor activation. Whether chemoreceptor neurons activation (like RTN) stimulate RVLM^C1^ neurons directly to coordinate cardiorespiratory coupling in conscious animals still a matter for further investigation.

## Perspectives and Future Directions: RVLM^C1^ and Hypertension

Because stimulation of the RVLM^C1^ neurons (or RVLM region) produces increase in sympathetic nerve activity and BP in rats, their chronic overactivation has been hypothesized to play a key role in the sympathetically-mediated hypertension [8, 42]. In fact, ablation of RVLM^C1^ neurons using the immuno toxin Dβh-Saporin attenuates the development of high blood pressure in SHR [43] and in salt-induced hypertension [44]. One important limitation is that this toxin, albeit, selective for catecholaminergic neurons, leads to ablation of Dβh+ neurons that also project to the RVLM. Therefore, it remains unclear whether the observed effects are mediated exclusively by the loss of RVLM^C1^ neurons or also involve the ablation of other catecholaminergic populations that innervate the RVLM. Future studies should try to investigate the specific role of RVLM^C1^ neurons in conscious, freely behaving rodents using selective methods of targeting and manipulation, especially acute loss-of-function of these neurons to avoid compensatory mechanisms and, whenever possible, measuring sympathetic activity along with BP in experimental models of hypertension. Another important aspect is to identify and distinguish RVLM^C1^ neurons subtypes and their role in the regulation of BP and systemic physiology. Intersectional approaches associated with single cell RNA information to target subpopulations of RVLM^C1^ should be incorporated into future experimental design.

The effect of RVLM^C1^ overactivation on driving hypertension may also be dependent on the nature and pattern of the stimuli. Recent evidence indicates that intermittent optogenetic stimulation of these neurons in conscious rats every 6 min for 1 h leads to last-longing hypertension up to one hour after the cessation of the stimuli [45]. We could speculate that this pattern of stimulation is analogous to intermittent exposure to stress or intermittent hypoxia, conditions also linked with elevated sympathetic activity and BP. Identifying the modality, intensity and pattern of stimuli and how this affects RVLM^C1^ neuron activity along with BP might illuminate mechanisms of cardiovascular disease.

The new findings on the importance of RVLM^C1^ neuron for the short-term regulation of BP offer a new perspective on the role of these neurons in pathophysiological states such as hypertension, suggesting that they may contribute not only to chronic, sustained sympathetic activation but also to the exaggerated, episodic cardiovascular responses observed in hypertension [46, 47]. For example, a recent study indicates that the sympathetic activation and cardiovascular responses occurring during spontaneous motor activity are greater in the SHR when compared to control [48]. This study suggests that the short-term BP responses are altered in SHR during physical activity. However, the neural mechanisms involved in this response are not known. Because the RVLM^C1^ neurons are activated during arousal and physical activity, it’s plausible to speculate that they might contribute to an enhanced sympathetic response to exercise in hypertension. Another example is the increased frequency of BP surges during REM sleep and other state-dependent cardiovascular changes in SHR [49, 50]. REM sleep is associated with sustained activation of RVLM^C1^ neurons, raising the possibility that altered recruitment of C1 neurons contributes to the enhanced frequency and magnitude of BP surges associated with hypertension. Because RVLM^C1^ neurons contribute to BP maintenance during postural changes, it is also possible that they might be directly or indirectly involved in the pathophysiology of orthostatic hypotension or supine hypertension present in neurodegenerative disease, such as multiple systems atrophy [51, 52].

It’s clear that the state-dependent nature of RVLM^C1^ neurons activity should also be considered when evaluating their role in the development of cardiovascular disease. Concomitant measurements of sleep-wake cycle and other natural behaviors should be incorporated to experimental design for a more powerful conclusion and to identify perhaps specific or subtle cardiovascular events in hypertension other than simply steady state BP. One interesting example is the BP surges that happens during sleep in obstructive sleep apnea patients [53, 54], a chronic disease that is strongly associated with hypertension [55]. The obstructive events during sleep leads to asphyxia and chemoreceptor activation culminating in arousal from sleep, increases in sympathetic activity and BP surges [54]. The role of RVLM^C1^ neurons in the acute cardiovascular responses during asphyxia-induced arousal remains unclear. In this context, one important challenge will be to dissociate the effect of arousal to the effect of asphyxia itself in the activation of RVLM^C1^ neurons in conscious rodents.

## Conclusion

**Fig. 1:**
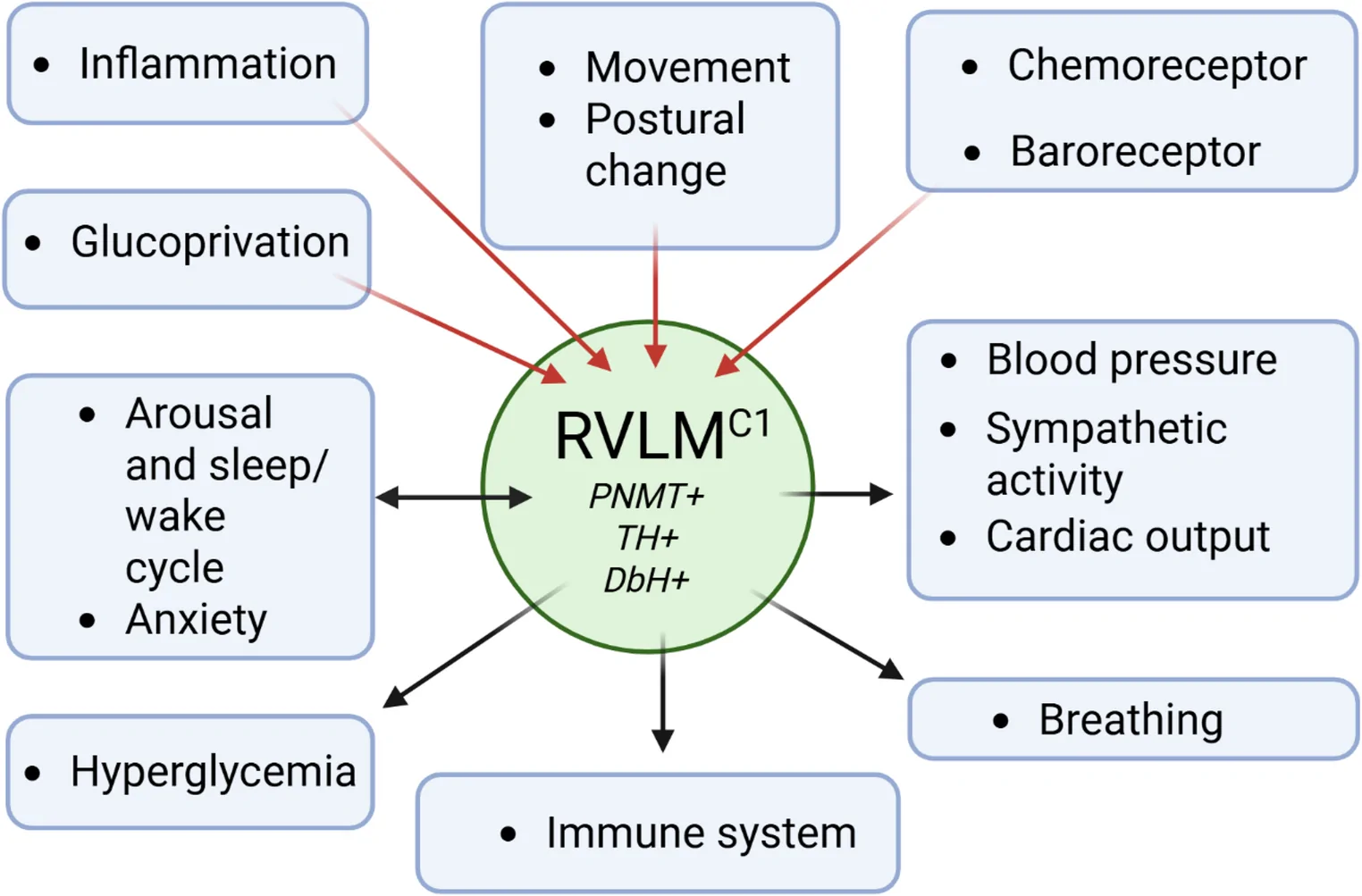
Polymodal activation of RVLM^C1^ neurons orchestrates adaptive physiological responses

Recent experimental approaches to recording and manipulating RVLM^C1^ neuron activity across different behaviors and physiological conditions have illuminated their role in cardiovascular homeostasis and several other aspects of systemic physiology in conscious, freely behaving animals (Fig. 1). Cardiometabolic diseases in both rodents and humans, including hypertension, are often accompanied by alterations in systemic physiology homeostasis, including hyperglycemia and metabolic dysfunction, inflammation, sleep–wake disruption and breathing abnormalities [55–57]. The complex interplay among these factors makes it challenging to parse out the mechanisms that contribute to disease progression. Given the established role of RVLM^C1^ neurons in the homeostatic and allostatic regulation of cardiovascular and systemic physiology, determining how these neurons contribute to cardiometabolic disease using novel experimental approaches represents an important and compelling challenge. Such studies may provide new insights into the neural mechanisms underlying hypertension and its associated pathophysiological states.

## Key References

- Souza GMPR, Thakkalapally H, Berry FE, Wisniewski LF, Atongazi UM, Stornetta DS, et al. Control of Blood Pressure Variability Across Behavioral States by Brainstem Adrenergic Neurons. *Circ Res.***2026** Feb 13;138 [4]. doi:https://doi.org/10.1161/CIRCRESAHA.125.326792.
  - ○This study used genetically-targeted fiber photometry to record from barosensitive RVLM^C1^ neurons during sleep-wake cycle and other natural behaviors in conscious rats, proving that these neurons have a dynamic activity across multiple states. Further, they shown that ablation of RVLM^C1^ neurons leads to increased blood pressure variability during wakefulness and postural changes. Altogether, this study indicates that RVLM^C1^ neurons are necessary for the control of blood pressure variability across dynamic behaviors in conscious animals.

- Fernández-Peña C, Pace RL, Fernando LM, Sheppard H, Pittman BG, Schwarz LA. Autonomic C1 neurons promote anxiety via activation of vlPAG. *Neuron.***2026** Jul; S0896627326004800. doi:https://doi.org/10.1016/j.neuron.2026.06.012.
  - ○This study used intersectional genetics methods to selectively target RVLM^C1^ neurons and study their function in behaving mice. They demonstrated that stimulation of RVLM^C1^ leads to anxiety-like states via specific projections to the ventral periaqueductal gray, revealing part of the circuitry sufficient and necessary for this response.

- Abe C, Katayama C, Bazek M, Nakamura Y, Ohbayashi K, Horii K, et al. Repeated activation of C1 neurons in medulla oblongata decreases anti-inflammatory effect via the hypofunction of the adrenal gland adrenergic response. Brain Behav Immun. **2023** Jul;111:138–50. doi:https://doi.org/10.1016/j.bbi.2023.04.003 PubMed PMID: 37037362.
  - ○This study demonstrates that RVLM^C1^ neurons play a critical role in regulating inflammation and protecting against acute lung injury through coordinated sympathetic and adrenal adrenergic responses. Importantly, repeated RVLM^C1^ activation impaired the adrenal adrenergic response and eliminated this protection, despite preserved plasma noradrenaline levels. These findings indicate that sustained adrenal adrenergic signaling driven by RVLM^C1^ neurons is essential for an effective anti-inflammatory response and protection from acute lung injury.

## Data Availability

No datasets were generated or analysed during the current study.
